# 512 Systemic Norepinephrine Impact on Tangential Split Thickness Skin Graft Outcomes in Burn Shock Patients

**DOI:** 10.1093/jbcr/irac012.143

**Published:** 2022-03-23

**Authors:** Ilina Terziyski, Albin A John, Annie Snitman, Grant Sorensen, Callie M Adams, Matteo Novello, John A Griswold

**Affiliations:** Texas Tech University Health Sciences Center, Lubbock, Texas; Texas Tech University Health Sciences Center, Lubbock, Texas; Texas Tech University Health Sciences Center, Lubbock, Texas; Texas Tech University Health Science Center, Lubbock, Texas; Texas Tech University Health Sciences Center, Lubbock, Texas; Texas Tech University Health Sciences Center, Lubbock, Texas; Texas Tech University Health Sciences Center, Lubbock, Texas

## Abstract

**Introduction:**

Blood pressure supporting agents like vasopressors are often used to treat patients with burn shock. Norepinephrine is part of the algorithms used by regional burn centers for fluid resuscitation in burn shock. In our population of burn shock patients, we have noticed an association of poor graft take when norepinephrine is used. We undertook this study to assess the relationship between systemic norepinephrine use and split-thickness skin graft (STSG) healing.

**Methods:**

We retrospectively identified burn patients who presented to our burn center from January 2014 –June 2020, who were treated with systemic vasopressors within the first 48 hours of admission, and received at least one tangential excision and STSG procedure as part of their treatment. We compared these patients to a matched control group of burn patients who did not receive vasopressors for resuscitative purposes. The primary outcome investigated was graft take percentage at time of graft takedown.

**Results:**

During the time frame, we found 19 patients and 19 matched controls within the same time period who did not receive norepinephrine. The mean percent graft take for patients treated with systemic norepinephrine was 77.9% (SE = 3.00), which was significantly lower than that of the control group, 92.8% (SE = 3.56) (P= < 0.001). Furthermore, patients who received norepinephrine had a statistically significant increase in both hospital (P= 0.038) and intensive care unit (ICU) length of stay (P= 0.009). The two populations were equivalent in all other characteristics such as TBSA, number of comorbidities, age, and resuscitation volumes.

**Conclusions:**

In this retrospective assessment, the use of norepinephrine seems to have a significant association with worse graft take and longer length of stay. Since graft loss begets more graft reoperations and a longer stay, our findings would lead one to incorporate norepinephrine as a last resort in the treatment algorithm for burn shock.

